# Late open conversion in ruptured abdominal aortic aneurysm after endovascular repair

**DOI:** 10.1590/1677-5449.008017

**Published:** 2018

**Authors:** Erol Kurç, Onur Sokullu, Serdar Akansel, Murat Sargın

**Affiliations:** 1 Dr Siyami Ersek Thoracic and Cardiovascular Surgery Training and Research Hospital, Department of Cardiovascular Surgery, Istanbul, Turkey.

**Keywords:** ruptured abdominal aortic aneurysm, EVAR, late open conversion, re-intervention, aneurisma roto da aorta abdominal, EVAR, conversão tardia para cirurgia aberta, reintervenção

## Abstract

Despite technological advances, the long-term outcomes of endovascular aortic aneurysm repair (EVAR) are still debatable. Although most endograft failures after EVAR can be corrected with endovascular techniques, open conversion may still be required. A 70-year-old male patient presented at the emergency unit with abdominal pain. Twice, in the third and fourth years after the first repair, a stent graft had been placed over a non-adhesive portion of the stent graft due to type Ia endoleaks. In the most recent admission, a CT scan showed type III endoleak and ruptured aneurysm sac. On this occasion the patient underwent late open conversion. The failure was repaired with total preservation of the main endovascular graft body and interposition of a bifurcated dacron graft. This case demonstrates that lifelong radiographic surveillance should be considered in this subset of patients. Late open conversion following EVAR of ruptured abdominal aortic aneurysms can be performed safely.

## Resumo

Apesar dos avanços tecnológicos, os desfechos de longo prazo do reparo endovascular de aneurismas da aorta abdominal (*endovascular aortic aneurysm repair* – EVAR) ainda são objeto de debate. Embora a maioria das falhas de endoenxerto após EVAR possam ser corrigidas com técnicas endovasculares, conversão para cirurgia aberta ainda pode ser necessária. Um paciente de 70 anos de idade, do sexo masculino, apresentou-se no serviço de emergência com dor abdominal. Duas vezes, dois e quatro anos após o primeiro reparo, um enxerto foi colocado sobre uma porção não adesiva do stent devido a endoleak tipo Ia. Na mais recente hospitalização, a tomografia computadorizada mostrou endoleak tipo III e ruptura de um saco aneurismático. Nesta ocasião, o paciente foi submetido a conversão tardia para cirurgia aberta. A falha foi tratada com preservação total do corpo principal do enxerto endovascular e interposição de um enxerto tipo Dacron bifurcado. Este caso demonstra que a vigilância radiográfica ao longo de toda a vida deveria ser considerada nesse subgrupo de pacientes. Conversão tardia para cirurgia aberta após EVAR de aneurismas rotos da aorta abdominal pode ser realizada com segurança.

## INTRODUCTION

 Improvements in endovascular stent technology and increasing experience and technical skills have resulted in endovascular aortic aneurysm repair (EVAR) becoming the first-choice treatment for patients with infrarenal abdominal aortic aneurysms. 

 Despite technological advances, only short-term outcomes have been shown to be better with EVAR than with open repair. Early advantages of EVAR are less pain, shorter hospitalization, rapid recovery, and significantly lower 30-day mortality and morbidity rates, 1 but it is also associated with a higher re-intervention rate than open repair. 2

 Indications for re-intervention include endoleaks, migration, graft disconnection, stent fractures, graft thrombosis, and infection. 3 Although most endograft failures after EVAR can be corrected with endovascular techniques, open conversion can still be required in 0.9 to 4.5% of cases, 4 and a specific rate of 2.1% is reported in the European Collaborators on Stent Graft Techniques for Aortic Aneurysm Repair (EUROSTAR) Registry. 2 Surgical re-intervention after failed EVAR is more challenging compared with primary open repair due to inflammation of the tissue surrounding the pre-existing stent graft. 

 In this report, we present a case of late open conversion with a partial stent-graft explantation technique for ruptured abdominal aortic aneurysm (rAAA) after a third failed EVAR. 

## CASE REPORT

**Figure 1:**
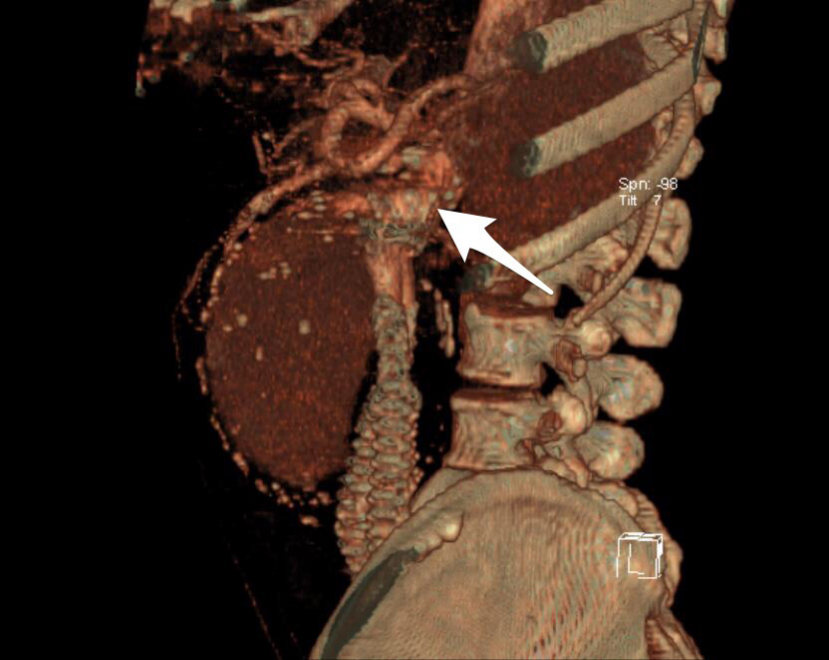
Reconstructed three-dimensional image shows a large, type Ia endoleak (arrow) and enlargement of the aneurysm to 120 mm in diameter, in the third year after the first repair.

 A 70-year-old, hypertensive, male patient presented at the emergency unit with a complaint of continuous crescendo abdominal pain. The patient had been treated 5 years previously with endovascular repair of a symptomatic infrarenal, fusiform, 119 mm diameter abdominal aortic aneurysm (AAA), at another institution. An initial surveillance scan at the 6-month follow-up, prior to admission, using computed tomography (CT) angiograms and abdominal plain radiographs, had shown no evidence of endoleak or sac enlargement. The patient had no further follow up until the third year after the first repair, and then, in the third and fourth years after the first repair, had been treated twice for stent migration and type Ia endoleak with placement of a stent graft over the non-adhesive portion of the stent graft ( Figure 1 ). 

**Figure 2:**
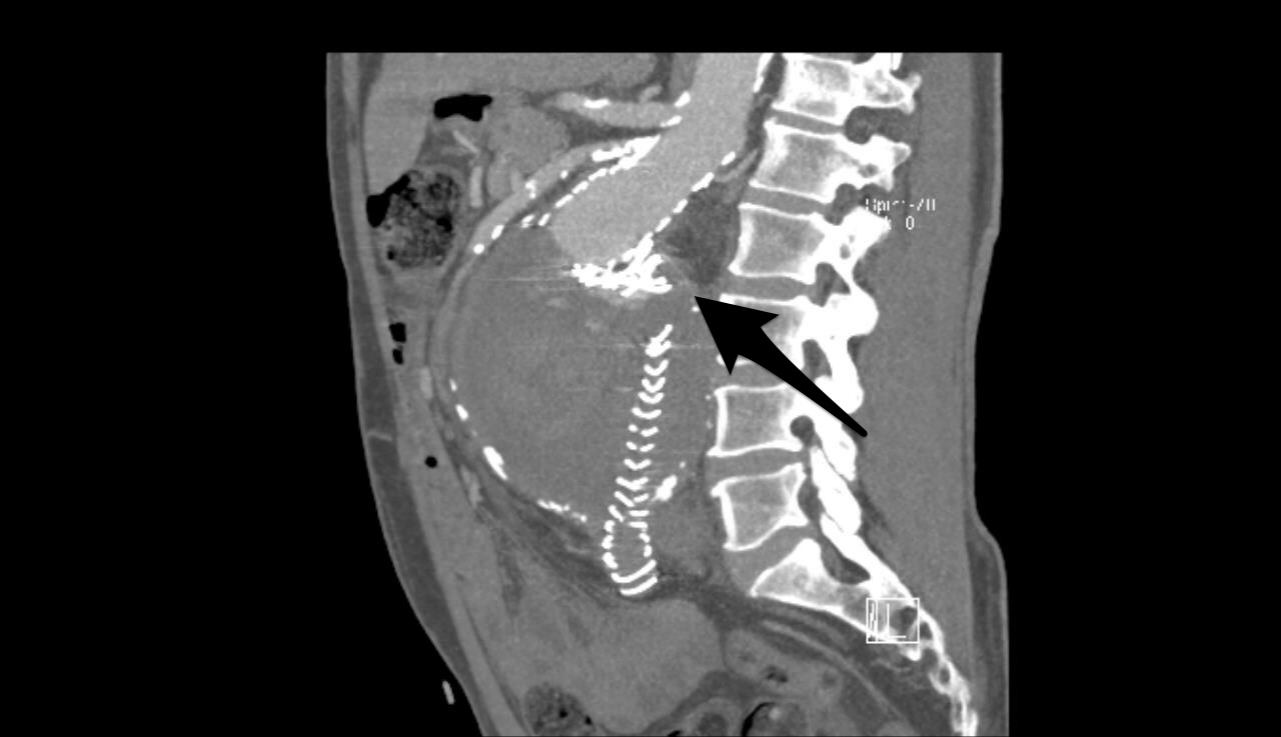
Post-procedure 62-month CT-scan depicting a large, type III endoleak (graft disconnection arrowed) and enlargement of the aneurysm to 133 mm in diameter.

 In the current admission, the patient had severe abdominal pain and hemodynamic instability. Expansion of the aneurysm sac (to 133 mm), type III endoleak (disconnection of main body from the bifurcated aortoiliac endovascular graft), and ruptured aneurysm sac were now evident on CT scan ( Figure 2 ). Open surgical treatment was planned for this patient, who was not suitable for endovascular repair because of hemodynamic instability and the anatomical difficulties caused by previous stent grafts. 

**Figure 3:**
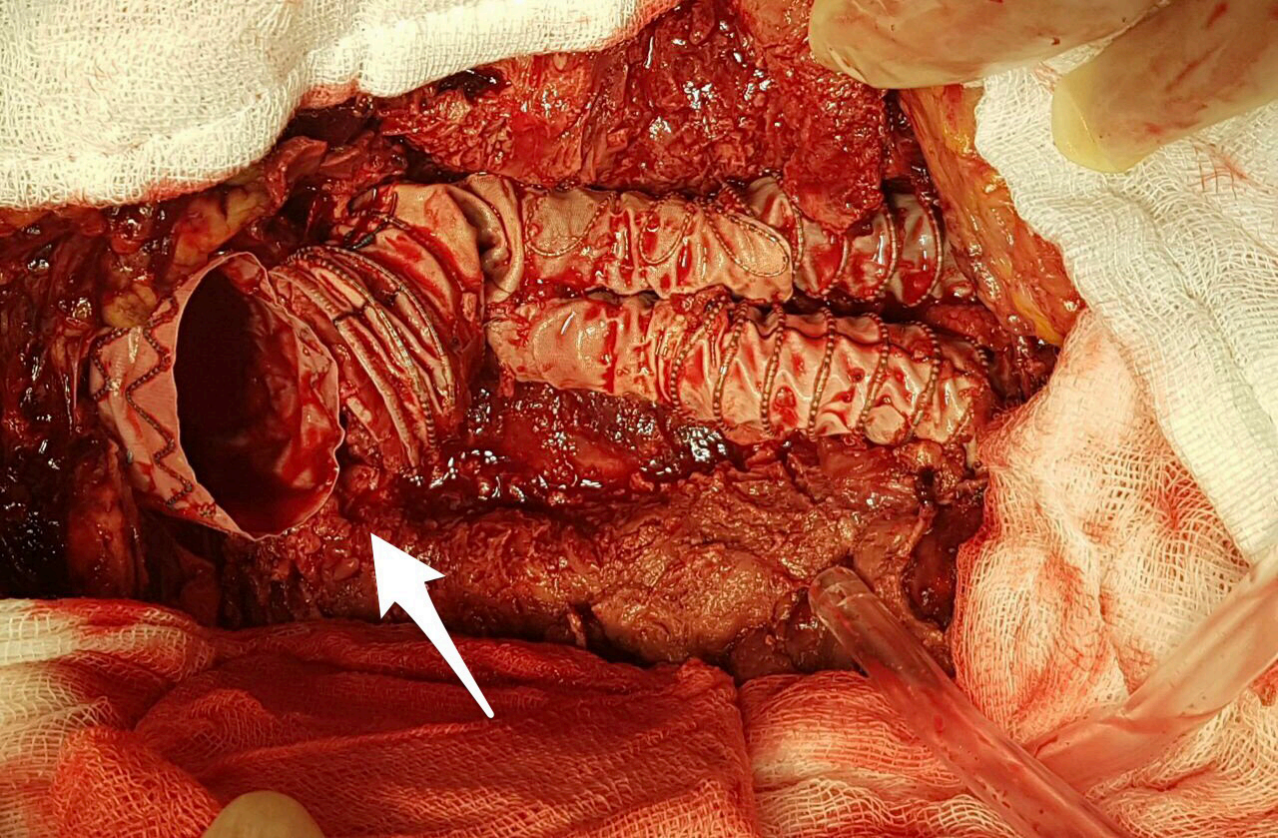
The aneurysm sac was opened under aortic cross-clamping. Disconnection of graft components is clearly visible (arrow).

 The patient underwent late open conversion through a midline transperitoneal approach. After dissecting the suprarenal neck and common iliac arteries, proximal aortic control was achieved by cross-clamping the suprarenal aorta and distal back-flow control was achieved by exposure and clamping of the iliac arteries below the stent graft. The aneurysm sac was opened by longitudinal aortotomy, a large quantity of mural thrombus was removed, and the disconnection of the main graft body from the bifurcated aortoiliac endovascular graft was observed ( Figure 3 ). The main stent graft body was preserved, since it was well-incorporated to the aortic wall and free from any leakage. Back-bleeding lumbar arteries were ligated. The proximal end of the bifurcated graft was sutured with 5-0 Gore-Tex suture to the main body of the endovascular graft. Distal anastomosis between aortic Dacron graft limbs and common iliac arteries was performed after partial removal of the distal iliac endograft limbs. 

 The patient was discharged uneventfully from the hospital on the 10th postoperative day. A control CT angiography performed six months after surgical treatment showed no new lesions. 

## DISCUSSION

 The process of aneurysm exclusion with EVAR, instead of surgery, offers some advantages including less pain, shorter hospitalization, rapid recovery, and significantly lower 30-day mortality and morbidity rates, especially in the high-risk patient. 1

 Despite technological advances, the risk of aneurysm sac enlargement and rupture after EVAR cannot be completely avoided and, as a result, it is associated with a higher re-intervention rate than open repair. Although most endograft failures after EVAR are corrected endovascularly, late conversion to open surgery has been reported widely. 

 In 2002, The EUROSTAR registry reported 4291 patients with EVAR between 1996 and 2002 and an annual rate for late conversion to open repair of 2.1% (elective and emergency) with a combined perioperative mortality rate of 43.6%. 2 In a recent review, late open conversion was reported to occur in 0.4-22% of patients who underwent EVAR, with an overall rate of 1.9%. 5 Forbes et al. reported that in their experience late open conversion occurred in 0.7% of patients following EVAR. 6

 Hölzenbein et al. 7 recommended endovascular redo interventions (aortic overstenting) as the first choice to correct an endoleak following EVAR. Conversion of an endovascular procedure into open vascular surgery is difficult and associated with high morbidity and mortality. 8 However, endovascular intervention may not be a favorable option for rupture after EVAR because of hemodynamic instability. Torsello et al. 9 reported a late open conversion case of AAA with rupture 16 months after treatment by an endograft. In a study reported by Schlösser et al., AAA rupture repair was performed in 160 patients by open conversion and in 26 using endovascular techniques. 10

 Late conversion to open surgery can be needed due to many reasons, including aneurysm enlargement, with or without endoleak, stent-graft migration or disconnection, thrombosis or infection of the stent graft, and aneurysm rupture with hemodynamic instability. 11 Current techniques of late open conversion include complete endograft preservation, partial endograft explantation, total explantation with in situ replacement or axillofemoral bypass. 12 The potential advantages of the partial preservation technique are lower risk of intraoperative injury to vessels and surrounding tissue, and shorter duration of aortic clamping and operation time. 12 Some authors offer a partial preservation technique for well-incorporated stent-graft components in the absence of stent-graft infection, because of the risk of bleeding resulting from aortic wall damage. 13

 Surgical exposure of the aneurysm can be achieved either through a transperitoneal or retroperitoneal approach. Both approaches have similar effectiveness in cases of late conversion. Another important element is the site of aortic cross-clamping. Infrarenal, suprarenal, or locations further above the fixation site (supraceliac clamping or balloon occlusion technique) may be preferred. 

 In the current case, graft disconnection and the ruptured aneurysm sac were shown by CT scan. We preferred open conversion to endovascular revision because of hemodynamic instability and the anatomical difficulty of the previous stent graft. Late open conversion was performed through a midline transperitoneal approach with total preservation of the main body of the endovascular stent-graft and interposition of a bifurcated aortic Dacron graft. 

 In conclusion, this case demonstrates that lifelong radiographic surveillance is crucial in this subset of patients. We advocate that following EVAR of ruptured abdominal aortic aneurysm, late open conversion with partial endograft explantation can be performed safely and successfully. 
